# Effects of Sevoflurane Exposure During Mid-Pregnancy on Learning and Memory in Offspring Rats: Beneficial Effects of Maternal Exercise

**DOI:** 10.3389/fncel.2018.00122

**Published:** 2018-05-03

**Authors:** Ziyi Wu, Xingyue Li, Yi Zhang, Dongyi Tong, Lili Wang, Ping Zhao

**Affiliations:** Department of Anesthesiology, Shengjing Hospital, China Medical University, Shenyang, China

**Keywords:** general anesthesia, mid-pregnancy, learning and memory, maternal exercise, brain-derived neurotrophic factor

## Abstract

Fetal exposure to general anesthetics may pose significant neurocognitive risks but methods to mitigate against these detrimental effects are still to be determined. We set out, therefore, to assess whether single or repeated *in utero* exposure to sevoflurane triggers long-term cognitive impairments in rat offspring. Since maternal exercise during pregnancy has been shown to improve cognition in offspring, we hypothesized that maternal treadmill exercise during pregnancy would protect against sevoflurane-induced neurotoxicity. In the first experiment, pregnant rats were exposed to 3% sevoflurane for 2 h on gestational (G) day 14, or to sequential exposure for 2 h on G13, G14 and G15. In the second experiment, pregnant rats in the exercise group were forced to run on a treadmill for 60 min/day during the whole pregnancy. The TrkB antagonist ANA-12 was used to investigate whether the brain-derived neurotrophic factor (BDNF)/TrkB/Akt signaling pathway is involved in the neuroprotection afforded by maternal exercise. Our data suggest that repeated, but not single, exposure to sevoflurane caused a reduction in both histone acetylation and BDNF expression in fetal brain tissues and postnatal hippocampus. This was accompanied by decreased numbers of dendritic spines, impaired spatial-dependent learning and memory dysfunction. These effects were mitigated by maternal exercise but the TrkB antagonist ANA-12 abolished the beneficial effects of maternal exercise. Our findings suggest that repeated, but not single, exposure to sevoflurane in pregnant rats during the second trimester caused long-lasting learning and memory dysfunction in the offspring. Maternal exercise ameliorated the postnatal neurocognitive impairment by enhancing histone acetylation and activating downstream BDNF/TrkB/Akt signaling.

## Introduction

A large number of studies in both humans and animals have demonstrated that exposure to anesthetic agents during neurodevelopment triggers neurodegeneration and subsequent long-term neurobehavioral abnormalities, such as learning and memory impairment (Andropoulos and Greene, 2017; Barton et al., 2018; Fodale et al., 2017). Most previous animal studies focused on neonates (especially rodents in the first week after birth since this is a period of rapid synaptogenesis, known as a brain growth spurt period), rather than on fetuses. It is, however, worth noting that the second trimester is a period of neurogenesis and neuronal migration (Palanisamy, 2012; Silbereis et al., 2016), during which the fetus is sensitive to the external environment. Furthermore, with the rapid development of laparoscopic procedures and fetal surgery, more and more pregnant women are undergoing surgeries during their pregnancy, especially in the second trimester, which are unrelated to the delivery. Taken together, these factors raise concern about the effects of currently used anesthetic agents on neurodevelopmental consequences for the fetus before birth. Indeed, published reports reveal conflicting experimental data about the effect of anesthetic agents on long-term behavioral changes in offspring and there are still great uncertainties and controversies in this field (Kong et al., 2012; Zheng et al., 2013; Suehara et al., 2016; Fang et al., 2017; Lee et al., 2017). Neonatal exposure to anesthetics induces neurodegenerative effects in a time-dependent manner (Murphy and Baxter, 2013; Amrock et al., 2015), which raises the possibility that the same might be true for fetal exposure.

Epigenetic mechanisms play a crucial role in shaping the developing brain and in cognitive processes. Abnormal histone acetylation has particular significance for neurocognitive impairments in some nervous system disorders (Jakovcevski and Akbarian, 2012; Bonnaud et al., 2016), such as postoperative cognitive dysfunction (Jia et al., 2015). Chromatin remodeling via histone acetylation is considered to play a vital role in reprogramming the expression of diverse genes, and may thus be involved in the changes in gene expression caused by general anesthetics. Brain-derived neurotrophic factor (BDNF) is a well-studied growth factor that acts as a key player in brain development and function. Up-regulation of BDNF expression has been shown to be driven by histone acetylation, and the result depends on the age and brain region (Bredy et al., 2007; Koppel and Timmusk, 2013; Martínez-Levy and Cruz-Fuentes, 2014; Chen and Chen, 2017). Thus, our first aim was to determine whether single or repeated *in utero* exposure to sevoflurane during the second trimester could produce changes in histone acetylation and expression of BDNF and in dendritic morphology and neurocognitive behavior.

Mounting evidence indicates that maternal exercise during gestation may favor fetal brain development and improve the cognitive performance of offspring (Robinson and Bucci, 2012). Enhanced learning ability and memory function have been shown to be associated with modifications in the structure and function of specific brain regions. It has also been consistently highlighted that a link between maternal exercise and enhanced expression of neurotrophins, such as BDNF, could, at least partially, account for the beneficial effects (Aksu et al., 2012; Gomes da Silva et al., 2016). However, the effects of maternal exercise on anesthesia-induced neurotoxicity in offspring remain unclear. Chromatin remodeling via histone acetylation is known to play a crucial role in gene expression regulation, and may thus be involved in the underlying mechanisms that contribute to changes in gene expression caused by exercise. In light of this evidence, the second aim of the present study was to investigate whether maternal treadmill exercise during pregnancy mitigates the putative detrimental effects of sevoflurane in prenatally exposed rats. To investigate the role of the BDNF signaling pathway in the maternal exercise effect, we used ANA-12, a selective antagonist of the tropomyosin receptor kinase B (TrkB), which is a BDNF receptor. Binding of BDNF activates TrkB, leading to activation of downstream signaling proteins, such as protein kinase B (also known as Akt), which are involved in the formation of dendritic spines (Majumdar et al., 2011; Nakai et al., 2014).

We thus aimed to test two hypotheses: (i) that repeated exposure to sevoflurane induces greater long-term cognitive impairment than single exposure, concomitant with decreased dendritic spine density, histone acetylation and BDNF expression; and (ii) that maternal treadmill exercise during gestation ameliorates these deleterious effects by enhancing histone acetylation and activating downstream BDNF/TrkB/Akt signaling.

## Materials and Methods

### Animals and Housing

Adult Sprague-Dawley rats were housed in a room maintained at 24 ± 1°C under a 12 h light/dark cycle, with free access to food and water. All experimental procedures and protocols were approved by The Laboratory Animal Care Committee of China Medical University, Shenyang, China (2016PS028K) and were performed in accordance with the Guidelines for the Care and Use of Laboratory Animals from the National Institutes of Health, USA. All efforts were made to minimize the total number of animals used and their suffering.

### Experimental Design and Exposure to Anesthetic

**Figure 1:**
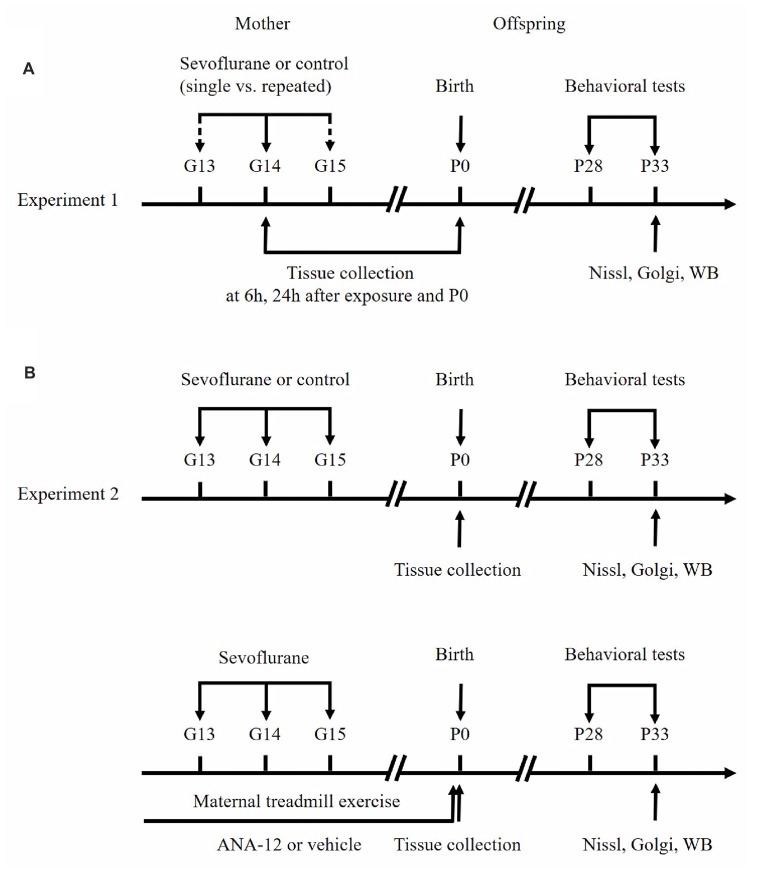
Schematic timeline of the experimental procedure. **(A)** First set of experiments. **(B)** Second set of experiments. See text for details.

The flow chart of the study protocol is presented in Figure 1. Three or four female rats were caged with one randomly selected male rat to enable free mating. On the second day, if vaginal emboli or sperm were detected by a vaginal smear, the female rat was considered to be at gestational day 0 (G0). For the first set of experiments (Figure 1A), pregnant rats were exposed to 3% (1.5 Minimum Alveolar Concentration [MAC]) sevoflurane in 30% oxygen in a specially constructed plastic chamber under one of four conditions (*n* = 9 per group): a single 2-h exposure (Sevo×1) or control condition (Ctrl×1) on G14, or three 2-h exposures (Sevo×3) or control condition (Ctrl×3), at G13, G14 and G15. Cesarean sections were performed 6 h and 24 h after anesthesia to extract fetal brain tissues for biochemical analysis (three dams/group at each time point). The remaining dams in each group (*n* = 3) were allowed to deliver naturally. Six offspring rats (two pups/dam) at postnatal day 0 (P0) were chosen from each group to be killed for removal of the hippocampus whilst the remaining pups continued to be reared. For the second set of experiments (Figure 1B), pregnant rats were randomly divided into four groups (*n* = 3 per group): Ctrl (Ctrl×3), Sevo (Sevo×3), Sevo + ME (Sevo×3 plus maternal exercise) and Sevo + ME + ANA-12 (Sevo×3 plus maternal exercise plus ANA-12). In the second experiments, all of the dams were allowed to deliver naturally. As in the first set of experiments, six offspring rats (two pups/dam) were chosen from each group at P0 to be sacrificed for removal of the hippocampus whilst the remaining pups continued to be reared. Concentrations of sevoflurane, oxygen and carbon dioxide in the chamber were measured using a gas monitoring device (Datex-Ohmeda Inc., Tewksbury, MA, USA). The rectal temperature of the dams was maintained at 37 ± 0.5°C. After exposure to anesthetic, all the dams were returned to their cages. The postnatal body weights of the rat pups were measured at P0, P7, P14 and P21, before behavior tests.

### Exercise Protocol

Pregnant rats in the exercise groups were forced to run on a treadmill for 60 min/day, 5 days per week, for a total of 3 weeks (from G0 until G20). The exercise load consisted of running at a speed of 8 m/min for 5 min, followed by 10 m/min for 25 min and then 12 m/min for the last 30 min. At G21, pregnant rats from all groups were maintained at rest (non-training) to monitor the birth of the litter.

### Inhibitors Administration

ANA-12 (Selleck Chemicals, Houston, TX, USA), a potent TrkB antagonist, was dissolved in corn oil containing 1% dimethyl sulfoxide, as previously described (Fan et al., 2016). ANA-12 (0.5 mg/kg, intraperitoneal, once daily), or vehicle, was administered to the pregnant dams for the whole period of the exercise paradigm.

### Western Blotting Analysis

Histone proteins were extracted using an EpiQuik™ total histone extraction kit (Epigentek, Farmingdale, NY, USA), according to the manufacturer’s protocol and western blotting was then performed as previously described (Jia et al., 2016). Total histone H3 antibody (1:2000; Cell Signaling Technology, Boston, MA, USA), acetyl-histone H3 (Lys9) antibody (ace-H3K9; 1:1000; Cell Signaling Technology, Boston, MA, USA), ace-H3K14 antibody (1:1000; Cell Signaling Technology) and ace-H3K27 antibody (1:1000; Cell Signaling Technology, Boston, MA, USA) were used to detect total H3, ace-H3K9, ace-H3K14 and ace-H3K27, respectively.

Fetal brain tissue and hippocampus homogenates were prepared for determination of BDNF, TrkB, p-TrkB, Akt, p-Akt, growth associated protein-43 (GAP43), postsynaptic density protein-95 (PSD95) and glyceraldehyde-3-phosphate dehydrogenase (GAPDH) expression with standard western blotting, as we previously described (Xu et al., 2016). Protein levels were determined by incubation with antibodies against BDNF (1:1000; Abcam, Cambridge, UK), TrkB (1:500; Abclonal, Woburn, MA, USA), p-TrkB (1:500; Abclonal), Akt (1:1000; Cell Signaling Technology, Boston, MA, USA), p-Akt (1:1000; Cell Signaling Technology, Boston, MA, USA), GAP43 (1:1000; Proteintech Biotechnology, Chicago, IL, USA), PSD95 (1:500; Proteintech Biotechnology, Chicago, IL, USA) and GAPDH (1:1000; Cell Signaling Technology, Boston, MA, USA). Protein bands were visualized by enhanced chemiluminescence and quantified with ImageJ software (NIH Image, Bethesda, MD, USA).

### Behavioral Tests

Rats were weaned at 3 weeks and caged in groups of three to five animals. Behavioral tests were carried out as previously described on days P28–P33, with minor modifications (Xu et al., 2016). To exclude the effects of estrous cycle on rodent behavior, behavioral tests were performed only on male offspring.

### Suspension Test

Suspension tests to evaluate motor outcomes were performed once a day, starting at 15:00 h, on days P28–P32. Twelve offspring rats (four from each dam) were forced to hold on to a 0.6 cm wide plastic level, placed 45 cm above the ground, with their anterior limbs. The test was over if: (i) the rat fell down; (ii) the suspension time exceeded 60 s; or (iii) the posterior limbs caught the level.

### Morris Water Maze (MWM) Tests

Morris water maze (MWM) tests were performed to test spatial learning and memory on days P28–P33. A round steel pool (diameter, 160 cm; depth, 60 cm), with a black wall and a removable cylindrical platform (diameter, 12 cm) in the second quadrant, was filled with warm water (20°C ± 1°C) to a level 1.5 cm above the top surface of the platform. The test comprised two phases: training sessions and a spatial searching test. Training sessions were carried out four times a day, starting at 8:00 am, for five consecutive days. Twelve offspring rats (four from each dam) were placed randomly into the water at different locations, facing the wall of the pool, to search for the platform. If a rat succeeded in finding the platform within 90 s, it was forced to remain on the platform for 20 s and the time taken to find the platform was defined as the escape latency. If a rat failed to find the platform within 90 s, it was gently guided to the platform and forced to stay there for 20 s. In this case, the escape latency was recorded as 90 s. In the spatial searching test, which was carried out on day 6, the platform was removed and the rat was released from the opposite quadrant and allowed to swim for 90 s. A video tracking system (Shanghai Mobile Datum Ltd, Shanghai City, China) recorded the swimming speed and escape latency. After each trial, the rat was dried and warmed using a heat lamp for 5 min before being returned to its regular cage.

### Nissl Staining

After the behavioral tests, three offspring rats (one from each dam) were randomly selected from each group. The rats were perfused transcardially with saline, followed by 4% paraformaldehyde, after which the brains were removed and post-fixed in 4% paraformaldehyde at 4°C overnight. Following paraffin embedding, coronal slices (4 μm thick) at approximately 3.3 mm caudal to bregma were obtained and stained with Nissl. Representative microphotographs of the pyramidal cell layer of the CA1 region were captured using a digital microscope camera. Images of cells from three Nissl-stained sections were calculated in each rat. Numbers of healthy, Nissl-positive, neuronal cells were counted by two individuals in a blinded manner with ImageJ software.

### Golgi Staining

After the behavioral tests, three offspring rats (one from each dam) were randomly selected from each group. Golgi staining was performed on 150 μm-thick frozen brain sections using a FD Rapid Golgi Stain Kit (FD NeuroTechnologies, Inc., Columbia, MD, USA), according to the manufacturer’s protocol. Spine density (spine number per 10 μm) for each neuron was analyzed with ImageJ software. The spines were counted on two or three segments of secondary dendrites.

### Statistical Analysis

All data were analyzed with SPSS 17.0 for Windows (SPSS Inc., Chicago, IL, USA) and are presented as mean ± S.E.M. All continuous variables were tested for assumption of normality using the Shapiro-Wilk test. If the assumption of normality was met, the Student’s *t*-test was used for data analysis in the first set of experiments, and one-way analysis of variance followed by Tukey *post hoc* multiple comparison tests was used in the second set of experiments. If the assumption of normality was unmet, the Mann-Whitney U test or Kruskal-Wallis H test was used, respectively. Results of maternal weight gain and escape latency were analyzed using two-way analysis of variance for repeated measurements. A *p* value < 0.05 was considered to be statistically significant. See Supplementary Table S1 for details.

## Results

### Effects of Prenatal Sevoflurane Exposure on Reproductive and Developmental Parameters

Reproductive and developmental data are shown in Table 1. Control and sevoflurane-exposed rat dams had approximately the same length of gestation and delivered similar numbers of pups. Sex ratio differences were not statistically significant. Neither single nor repeated exposure to sevoflurane had a significant effect on body weights of offspring on P1, P7, P14 and P21.

**Table 1:** Reproductive and developmental parameters.

Parameter	Ctrl×1	Sevo×1	Ctrl×3	Sevo×3
No. of dams	3	3	3	3
Gestation length (days)	21	21	21	21
Litter size	11.33 ± 1.76	12.33 ± 1.86	11.33 ± 1.86	11.33 ± 1.20
Male/female ratio	1.02 ± 0.18	1.05 ± 0.19	1.18 ± 0.41	1.02 ± 0.19
Body weight (g)				
P1	7.03 ± 0.03	7.03 ± 0.02	7.08 ± 0.02	7.07 ± 0.23
P7	14.83 ± 0.07	15.00 ± 0.06	14.93 ± 0.05	14.97 ± 0.06
P14	34.82 ± 0.35	35.16 ± 0.23	35.35 ± 0.28	35.00 ± 0.35
P21	50.94 ± 0.49	50.63 ± 0.42	50.65 ± 0.38	50.30 ± 0.38

*Data are presented as mean ± S.E.M. The body weights at P1 considered the whole litter (male and female pups). The body weight at P7, P14 and P21 considered the body weights of male rats only. Independent *t*-test was used for data analysis*.

### Repeated Maternal Exposure to Sevoflurane Induced Changes in Histone Acetylation and BDNF Levels in Fetal and Offspring Rats

**Figure 2:**
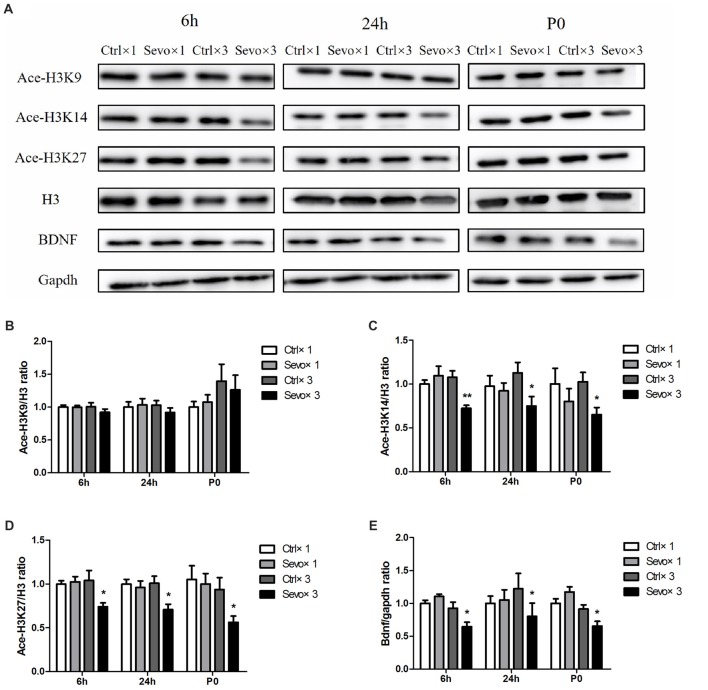
Effects of fetal exposure to sevoflurane on expression of acetylated histones H3 in the fetal brain tissues or hippocampus. Fetal brain tissues were harvested at two time points: 6 h and 24 h after anesthesia. Hippocampus was harvested at P0. **(A)** Representative western blotting images. **(B)** Quantitative analysis of ace-H3K9. **(C)** Quantitative analysis of ace-H3K14. **(D)** Quantitative analysis of ace-H3K27. **(E)** Quantitative analysis of brain-derived neurotrophic factor (BDNF). H3 or gapdh was run as an internal standard. Values are mean ± S.E.M (*n* = 6). *p < 0.05, compared with Ctrl×3 group; **p < 0.01, compared with Ctrl×3 group. Independent *t*-test, Welch’s *t*-test, or Mann-Whitney U test was used for data analysis.

Repeated exposure to sevoflurane significantly reduced levels of ace-H3K14 (Figure 1A, *p* < 0.01, *p* < 0.05, *p* < 0.05 at 6 h and 24 h after anesthesia and P0, respectively), ace-H3K27 (Figures 2A,D, *p* < 0.05, *p* < 0.05, *p* < 0.05 at 6 h and 24 h after anesthesia and P0, respectively) and BDNF protein (Figures 2A,E, *p* < 0.05, *p* < 0.05, *p* < 0.05 at 6 h and 24 h after anesthesia and P0, respectively) in fetal brain tissue 6 h and 24 h after sevoflurane anesthesia and in postnatal hippocampus at P0, compared with the Ctrl×3 group. There was, however, no significant difference between the Ctrl×1 group and the Sevo×1 group (Figures 2A,C–E). Of note, ace-H3K9 levels were not significantly changed after either single or repeated exposure to sevoflurane (Figures 2A,B), indicating that ace-H3K9 was not involved in sevoflurane-induced neurotoxicity in this maternal fetal rat model.

### Repeated Maternal Exposure to Sevoflurane Caused Learning and Memory Impairment in Offspring Rats

There were no significant differences in the results of suspension tests between pups born to dams in the control and sevoflurane-exposed groups (Supplementary Figure S1). Suspension times were all ~35 s, indicating that there was no motor function impairment in either the Sevo×1 group or the Sevo×3 group.

**Figure 3:**
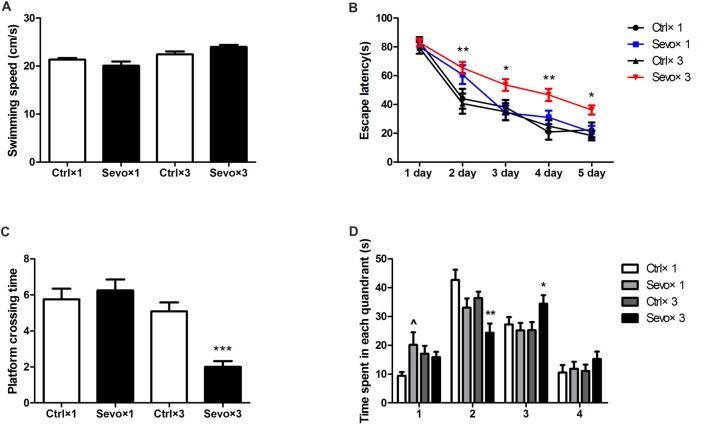
Morris water maze tests of the learning and memory function tests. **(A)** The average swimming speed. **(B)** Escape latency. **(C)** Platform crossing time. **(D)** Time spent in each quadrant. These tests were performed during P28–33 (*n* = 12/group). Values are mean ± S.E.M. *p < 0.05, compared with Ctrl×3 group; **p < 0.01, compared with Ctrl×3 group; ***p < 0.001, compared with Ctrl×3 group; ^∧^*p* < 0.05, compared with Ctrl×1 group. Independent *t*-test was used to determine the differences of the average swimming speed. Two-way analysis of variance for repeated measurements followed by Tukey post test was used to determine the difference of escape latency. Mann-Whitney U test was used to determine the difference of platform crossing times. Independent *t*-test or Mann-Whitney U test was used to determine the difference of time spent in each quadrant.

There were also no statistically significances in swimming speed during the MWM test (Figure 3A), and escape latency did not differ between the Ctrl×1 group and the Sevo×1 group in the orientation navigation part of the MWM tests (Figure 3B). Compared with rats in the Ctrl×3 group, however, rats in the Sevo×3 group needed more time to find the platform (Figure 3B, *p* < 0.01, *p* < 0.05, *p* < 0.05, *p* < 0.01 at 2nd, 3rd, 4th, and 5th day, respectively). In the spatial searching test on day 6, platform-crossing times (Figure 3C) and time spent in the target quadrant (quandrant 2; Figure 3D) did not differ between the Ctrl×1 group and the Sevo×1 group. However, compared with rats in the Ctrl×3 group, rats in the Sevo×3 group made less platform crossings (Figure 3C, *p* < 0.001) and spent less time in the target quadrant (quandrant 2; Figure 3D, *p* < 0.01).

### Repeated Maternal Exposure to Sevoflurane Reduced Expression Levels of Synaptic Markers in the Hippocampus of Offspring Rats

**Figure 4:**
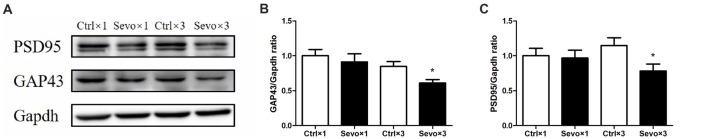
Effects of fetal exposure to sevoflurane on expression of PSD95 and GAP43 in the hippocampus of offspring rats at P33. Hippocampus was harvested at P33 after the behavior tests (*n* = 6/group). **(A)** Representative western blotting images. **(B)** Quantitative analysis of PSD95. **(C)** Quantitative analysis of GAP43. Gapdh was run as an internal standard. Values are mean ± S.E.M. *p < 0.05, compared with Ctrl×3 group. Independent *t*-test was used for data analysis.

The effects of prenatal sevoflurane anesthesia on levels of synaptic markers GAP43 and PSD95 in the hippocampus of offspring rats at P33 were determined by western blot. Quantification of the western blots showed that repeated sevoflurane anesthesia in pregnant rats significantly reduced levels of GAP43 (Figures 4A,B, *p* < 0.05) and PSD95 (Figures 4A,C, *p* < 0.05) compared with those in the Ctrl×3 group. There was, however, no significant difference between the Ctrl×1 group and the Sevo×1 group (Figure 4). It is worth noting that GAP43 (Supplementary Figures S2A,B, *p* < 0.05) and PSD95 (Supplementary Figures S2A,C, *p* < 0.05) levels were decreased in Sevo×3 group at 6 h after anesthesia, compared with Ctrl×3 group. The reduction of synaptic markers has been shown to be associated with decreases in synapse number or synaptic loss. Our results are consistent with the notion that anesthetic-induced reduction in GAP43 and PSD 95 may lead to decreased neuroplasticity and cognitive impairments in developing brain (Wang et al., 2012; Zhang et al., 2015; Lu et al., 2017).

### Repeated Maternal Exposure to Sevoflurane Caused Neuronal Cell Loss and Reduced Dendritic Spine Density in the Hippocampal CA1 Region of Offspring Rats

**Figure 5:**
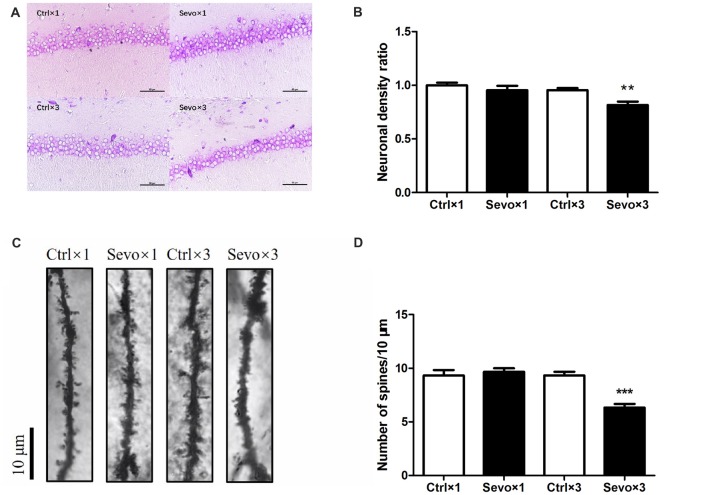
Histopathological examination. Nissl staining and Golgi staining were performed after behavior tests (*n* = 3). **(A)** Nissl staining in the CA1 region of hippocampus under ×400. **(B)** Neuronal density ratio changes. **(C)** Golgi staining in the CA1 of hippocampus under ×1000. **(D)** Histograms represented the number of dendritic spines/10 μm. Values are mean ± S.E.M. **p < 0.01, compared with Ctrl×3 group; ***p < 0.001, compared with Ctrl×3 group. Independent *t*-test was used for data analysis.

The hippocampus, especially the CA1 region, is associated with learning and memory. Because of this, we used Nissl staining to study hippocampal architecture after the behavioral tests. Nissl staining was used to distinguish viable neurons from apoptotic or necrotic neurons. Viable cells exhibited abundant cytoplasm and Nissl substance (stained as dark blue), readily distinguishable oval nuclei (stained as light blue) and prominent nucleoli, while apoptotic or necrotic cells exhibited pyknotic morphology, with amorphous or fragmented nuclei. There was a significant difference in cell density in the CA1 region between the Ctrl×3 group and the Sevo×3 group (Figures 5A,B, *p* < 0.01), but there was no significant difference between the Ctrl×1 group and the Sevo×1 group (Figures 5A,B).

Fully impregnated CA1 pyramidal cells were detected by Golgi staining, and the spines of the secondary dendrites (Figure 5C) were visualized with a light microscope with a 100× oil immersion objective lens. Only the density of the dendritic spines was determined since the different types of spine (e.g., thin, mushroom or branched dendrites) were not always clearly distinguishable. The spine density in the Sevo×3 group was significantly reduced compared with that in the Ctrl×3 group (Figures 5C,D, *p* < 0.001), but there was no significant difference between the Ctrl×1 group and the Sevo×1 group (Figures 5C,D).

### Maternal Weight Gain During Pregnancy

All pregnant rats were weighed every 2 days from G1 to G21, between 07:00 h and 08:00 h. The pregnant rats in the exercise group were always weighed before the physical training sessions. Animals in all groups gained weight during pregnancy, with no significant differences between the groups (Supplementary Figure S3).

### Maternal Exercise Ameliorated Sevoflurane-Induced Reduction of Histone Acetylation and BDNF Expression

**Figure 6:**
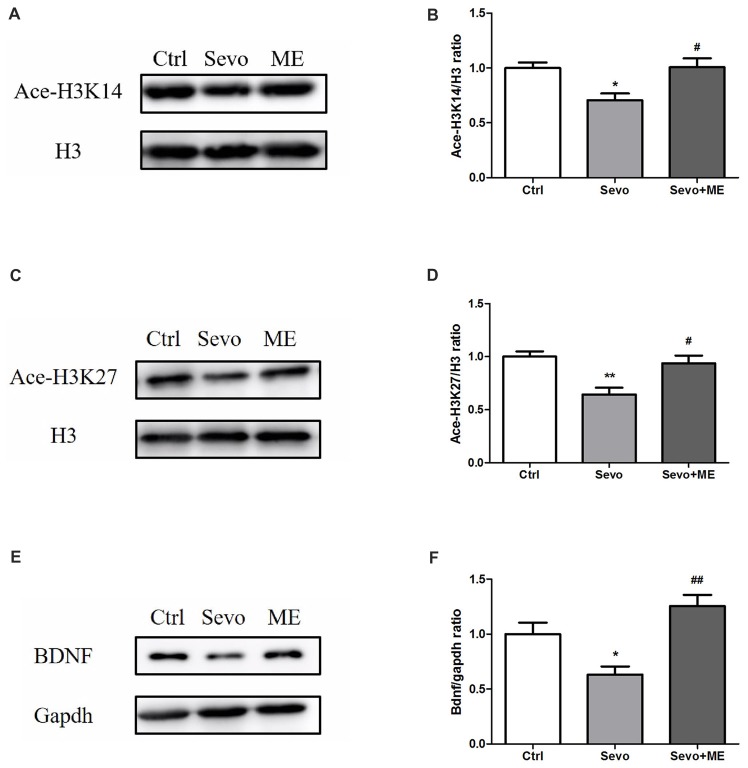
Sevoflurane-induced reduction in histone acetylation and BDNF was attenuated by maternal exercise. Hippocampus was harvested at P0 (*n* = 6). **(A)** Representative western blotting images of ace-H3K14. **(B)** Quantitative analysis of ace-H3K14. **(C)** Representative western blotting images of ace-H3K27. **(D)** Quantitative analysis of H3K27. **(E)** Representative western blotting images of BDNF. **(F)** Quantitative analysis of BDNF. H3 or gapdh was run as an internal standard. Values are mean ± S.E.M. *p < 0.05, compared with Ctrl group; **p < 0.01, compared with Ctrl group; ^#^*p* < 0.05, compared with Sevo group; ^##^*p* < 0.01, compared with Sevo group. One-way analysis of variance followed by Tukey *post hoc* multiple comparison tests was used for data analysis.

To determine whether maternal exercise affected histone acetylation and BDNF expression, hippocampi were harvested at P0. Sevoflurane-induced reduction in ace-H3K14, ace-H3K27 and BDNF was attenuated by maternal exercise (Figure 6, Ctrl vs. Sevo: *p* < 0.05, *p* < 0.01, *p* < 0.05; Sevo + ME vs. Sevo: *p* < 0.05, *p* < 0.05, *p* < 0.01; for ace-H3K14, ace-H3K27 and BDNF, respectively). There were no significant defferences between the Ctrl group and ME + Sevo group. Since BDNF is known to affect learning and memory, these results suggest a possible role of epigenetic regulation of BDNF in the effects of sevoflurane and maternal exercise on neurodevelopment and cognition.

### Beneficial Effects of Maternal Exercise in Reducing Sevoflurane-Induced Learning and Memory Impairment Were Blocked by TrkB Inhibition

**Figure 7:**
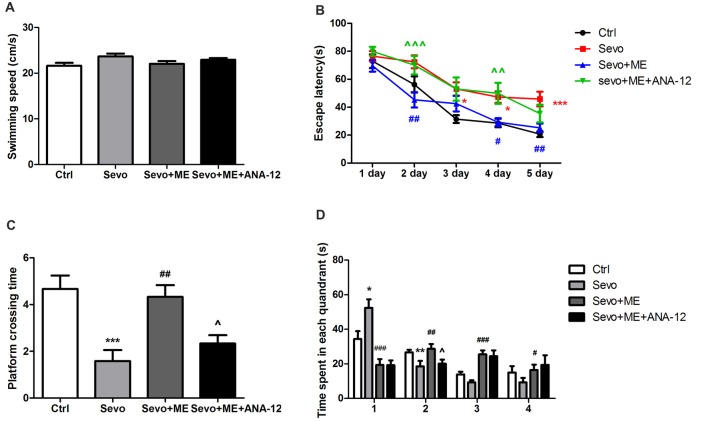
Maternal exercise ameliorated sevoflurane-induced learning and memory impairment, blocked by TrkB inhibition.** (A)** The average swimming speed. **(B)** Escape latency. **(C)** Platform crossing time. **(D)** Time spent in each quadrant. These tests were performed during P28–33 (*n* = 12/group). Values are mean ± S.E.M. *p < 0.05, compared with Ctrl group; **p < 0.01, compared with Ctrl group; ***p < 0.001, compared with Ctrl group; ^#^*p* < 0.05, compared with Sevo group; ^##^*p* < 0.01, compared with Sevo group; ^###^*p* < 0.001, compared with Sevo group; ^∧^*p* < 0.05, compared with Sevo + ME group; ^∧∧^*p* < 0.01, compared with Sevo + ME group; ^∧∧∧^*p* < 0.001, compared with Sevo + ME group. Kruskal-Wallis H test was used to determine the differences of the average swimming speed. Two-way analysis of variance for repeated measurements followed by Tukey post test was used to determine the difference of escape latency. Two-way analysis of variance for repeated measurements followed by Tukey post test was used to determine the difference of Platform crossing times. Two-way analysis of variance for repeated measurements followed by Tukey post test or Kruskal-Wallis H test was used to determine the difference of time spent in each quadrant.

No statistically significant differences were observed in swimming speed during the MWM test (Figure 7A). Consistent with the first set of experiments on learning and memory, repeated exposure to sevoflurane had a significant effect on escape latency (Figure 7B, *p* < 0.05, *p* < 0.05, *p* < 0.001 at 3rd, 4th, and 5th day, respectively), platform-crossing times (Figure 7C, *p* < 0.001) and time spent in the target quadrant (quandrant 2; Figure 7D, *p* < 0.01). Maternal exercise** s**uccessfully shortened escape latency during the training sessions of the MWM test (Figure 7B, *p* < 0.01, *p* < 0.05, *p* < 0.01 at 2nd, 4th, and 5th day, respectively) and increased platform-crossing times (Figure 7C, *p* < 0.01) and time spent in the target quadrant (quandrant 2; Figure 7D, *p* < 0.01) on day 1 after the training sessions. There were no significant differences between the Ctrl group and ME + Sevo group in escape latency and platform-crossing times and time spent in the target quadrant (quandrant 2). However, treatment with ANA-12 reversed the protective effect of maternal exercise (Figures 7B–D, escape latency: *p* < 0.001, *p* < 0.001 at 2nd and 4th day, respectively; platform crossing times: *p* < 0.05; time spent in the target quandrant (quandrant 2): *p* < 0.05), suggesting that TrkB signaling may be involved in the beneficial effects of maternal exercise on cognition in offspring rats after repeated exposure to sevoflurane.

### Ability of Maternal Exercise to Ameliorate Sevoflurane-Induced Neuronal Cell Loss and Reduction in Dendritic Spine Density Was Blocked by TrkB Inhibition

**Figure 8:**
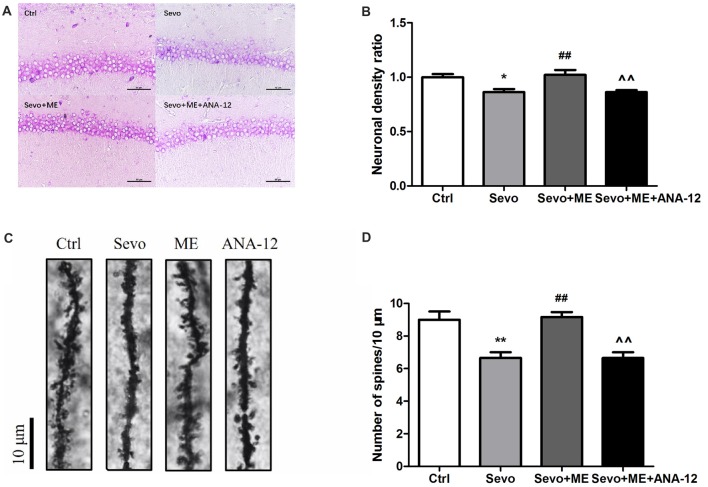
Maternal exercise ameliorated sevoflurane-induced neuronal cell loss and decreased dendritic spine density, blocked by TrkB inhibition. Nissl staining and Golgi staining were performed after behavior tests (*n* = 3).** (A)** Nissl staining in the CA1 region of hippocampus under ×400. **(B)** Neuronal density ratio changes. **(C)** Golgi staining in the CA1 of hippocampus under ×1000. **(D)** Histograms represented the number of dendritic spines/10 μm. Values are mean ± S.E.M. *p < 0.05, compared with Ctrl group; **p < 0.01, compared with Ctrl group; ^##^*p* < 0.01, compared with Sevo group; ^∧∧^*p* < 0.01, compared with Sevo + ME group. One-way analysis of variance followed by Tukey *post hoc* multiple comparison tests was used for data analysis.

Sevoflurane significantly reduced the number of neuronal cells that stained positively with Nissl (Figures 8A,B, *p* < 0.05) and the number of dendritic spines detected by Golgi staining (Figures 8C,D, *p* < 0.01) in the CA1 region of hippocampus. These effects were ameliorated by maternal exercise (Figure 8, Nissl: *p* < 0.01; Golgi: *p* < 0.01) but the beneficial effect of exercise was blocked by treatment with ANA-12 (Figure 8, Nissl: *p* < 0.01; Golgi: *p* < 0.01). There were no significant differences between Ctrl group and Sevo + ME group.

### Maternal Exercise Ameliorated Sevoflurane-Induced Reduction in Expression of Synaptic Markers and This Effect Was Blocked by TrkB Inhibition

**Figure 9:**
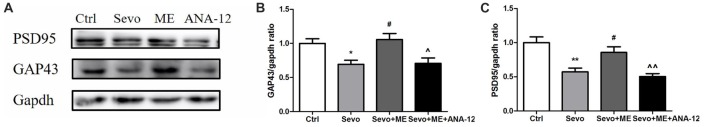
Maternal exercise ameliorated sevoflurane-induced reduction of synaptic markers expression, blocked by TrkB inhibition. Hippocampus was harvested at P33 after the behavior tests (*n* = 6).** (A)** Representative western blotting images. **(B)** Quantitative analysis of GAP43. **(C)** Quantitative analysis of PSD95. Gapdh was run as an internal standard. Values are mean ± S.E.M. *p < 0.05, compared with Ctrl group; **p < 0.01, compared with Ctrl group; ^#^*p* < 0.05, compared with Sevo group; ^∧^*p* < 0.05, compared with Sevo + ME group; ^∧∧^*p* < 0.01, compared with Sevo + ME group. One-way analysis of variance followed by Tukey *post hoc* multiple comparison tests was used for data analysis.

Because repeated exposure to sevoflurane decreased levels of GAP43 and PSD95 in the hippocampus of offspring, we next determined the effects of maternal exercise on synaptic markers. Maternal exercise mitigated sevoflurane-induced decreases in levels of GAP43 and PSD95 in the hippocampus of offspring rats at P33 (Figure 9, Ctrl vs. Sevo: *p* < 0.05, *p* < 0.01; Sevo + ME vs. Sevo: *p* < 0.05, *p* < 0.05 for GAP43 and PSD95, respectively), but this effect was blocked by TrkB inhibition (Figure 9, Sevo + ME + ANA-12 vs. Sevo + ME: *p* < 0.05, *p* < 0.01 for GAP43 and PSD95, respectively). There were no significant differences between Ctrl group and Sevo + ME group.

### TrkB Inhibition Blocked Maternal Exercise-Induced Activation of Trkb and Akt

**Figure 10:**
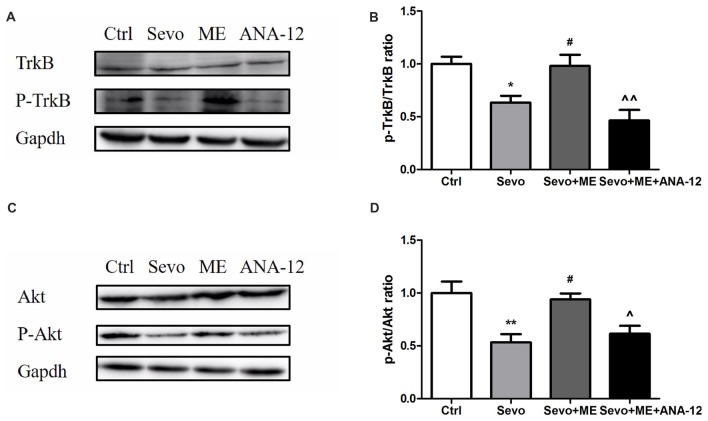
Maternal exercise ameliorated sevoflurane-induced reduction of P-TrkB/TrkB and P-Akt/Akt, blocked by TrkB inhibition. Hippocampus was harvested at P0 (*n* = 6). **(A)** Representative western blotting images of P-TrkB and TrkB. **(B)** Quantitative analysis of P-TrkB/TrkB. **(C)** Representative western blotting images of P-Akt and Akt. **(D)** Quantitative analysis of P-Akt/Akt. Gapdh was run as an internal standard. ^∧∧^*p* < 0.01, compared with Sevo + ME group. Values are mean ± S.E.M (*n* = 6). *p < 0.05, compared with Ctrl group; **p < 0.01, compared with Ctrl group; ^#^*p* < 0.05, compared with Sevo group; ^∧^*p* < 0.05, compared with Sevo + ME group. One-way analysis of variance followed by Tukey *post hoc* multiple comparison tests was used for data analysis.

As well as reducing levels of BDNF, sevoflurane also reduced phosphorylation/activation of TrkB (Figures 10A,B, *p* < 0.05) and Akt (Figures 10C,D, *p* < 0.01). This reduction was attenuated by maternal exercise (Figure 10, *p* < 0.05, *p* < 0.05 for p-TrkB/TrkB and p-Akt/Akt, respectively). ANA-12 blocked the effects of maternal exercise on the phosphorylation/activation of TrkB (Figures 10A,B, *p* < 0.01) and Akt (Figures 10C,D, *p* < 0.05). ANA-12 thus inhibited maternal exercise-induced activation of the TrkB signaling pathway in the hippocampus of offspring rat after repeated prenatal exposure to sevoflurane. There were no significant differences between Ctrl group and Sevo + ME group.

## Discussion

The principal finding of the present study is that repeated, but not single, exposure of fetal rats to sevoflurane in the second trimester induces long-term cognitive impairment that is accompanied by decreased histone acetylation and reduced levels of BDNF in fetal brain tissue or hippocampus. Maternal exercise during the whole pregnancy prevented these cognitive impairments. The mechanisms of the protective effect may be related to restoration of histone acetylation and downstream BDNF/TrkB/Akt signaling cascades.

Although the effects of exposure to sevoflurane during the fetal period on long-term neurodevelopmental outcomes have been investigated previously, the results are conflicting and controversial. For example, in one earlier study, a single 2-h exposure to 2.5% sevoflurane in pregnant mice impaired learning and memory function in offspring mice (Zheng et al., 2013). In another study, however, which used exactly the same protocol for anesthesia, neither single nor repeated exposure to sevoflurane in pregnant mice caused long-lasting behavioral abnormalities in offspring mice (Lee et al., 2017). The discrepancies between the studies may be caused by species differences, differences in anesthetic protocol (exact gestational age, type of anesthetic agent, dose and duration of exposure) or the sensitivity and procedures of the behavioral tests. Consistent with many other studies, we exposed pregnant rats to sevoflurane at G13–G15, an age that is assumed to correspond to the second trimester of human pregnancy (Workman et al., 2013). We chose to expose the pregnant rats to 3% (1.5 MAC) sevoflurane for 2 h since this length of time is more relevant to clinical situations. In some cases, however, to relax uterine smooth muscle and provide adequate anesthesia for fetal interventions, fetal brains can be exposed to higher concentrations (e.g., 1.0–1.5 MAC) of anesthetic than those that are commonly used in clinical practice (Olutoye et al., 2016; Wang et al., 2018). Some surgical procedures, such as fetal surgery to correct birth defects, may also need to be repeated (Olutoye et al., 2016). In fact, evidence from both preclinical and retrospective studies showed that the risk of neurotoxicity is increased by repeated, but not single, exposures to anesthetic agents (Andropoulos and Greene, 2017). We, therefore, compared the long-term effects of one and three episodes of sevoflurane anesthesia during the fetal developmental period. Behavioral tests showed that a single 2-h exposure to sevoflurane in mid-gestation caused no learning and memory impairment. Three 2-h exposures to sevoflurane did, however, have a detrimental effect on learning and memory function, in the absence of surgery or other associated environmental, parental and gestational factors that cannot be controlled for in clinical studies. The behavioral test results were confirmed by Nissl and Golgi staining.

Epigenetic dysfunction has been shown to be strongly associated with abnormal brain function and neurodegenerative diseases (Qureshi and Mehler, 2011; Jakovcevski and Akbarian, 2012). The phenotypes and pathogenesis of anesthesia-induced developmental neurotoxicity, such as degenerative changes and cognitive deficits, are similar to those seen in neurodegenerative diseases. Histone acetylation is a common type of epigenetic modification that is widely involved in the shaping of the developing brain and long-term learning and memory adaptation caused by environmental stimuli. Histone acetylation remodels the “epigenome”, resulting in changes in the expression of diverse genes and in neural function (Bonnaud et al., 2016). In general, increased histone acetylation is associated with active gene transcription, whereas histone deacetylation negatively regulates gene transcription. Extensive studies have linked increased histone-tail acetylation (such as H3K9, H3K14 and H3K27) to facilitated learning and memory, while dysregulation of histone acetylation contributes to cognitive impairment by suppressing key learning and memory genes (Peixoto and Abel, 2013). It has been previously reported that neonatal exposure of rodents to general anesthetics initiates changes in histone acetylation in the brain (Jia et al., 2016; Liang and Fang, 2016). Consistent with this study, we found that repeated fetal exposure to sevoflurane, but not single exposure, reduced histone acetylation at H3K14 and H3K27. Of note, in contrast to a previous study based on neonatal rat pups (Jia et al., 2016), we found that ace-H3K9 was not changed after either single or repeated exposure to anesthetic. Not all lysine residues in histone proteins are associated with the development of neurodegenerative disorders and different diseases might induce changes in the level of histone acetylation at different sites. This suggests that investigating these specific acetylation sites through genome-wide analysis of epigenetic modifications would help to elucidate the mechanisms underlying anesthesia-induced neurotoxicity. To clarify how altered histone acetylation leads to cognitive deficits in offspring rats, we investigated the expression of proteins related to cognitive performance. BDNF, a neurotrophin that is involved in synapse regulation, is a target of histone acetylation. In our study, BDNF was decreased in both fetal brain tissue and postnatal hippocampus after repeated, but not single, exposure to sevoflurane. Levels of synaptic plasticity-related proteins, PSD95 and GAP43, were downregulated only after repeated exposure. PSD95 and GAP43 levels are possibly regulated by BDNF, since numerous studies have described the dominant role of BDNF in synaptic plasticity in the hippocampus (Kowianski et al., 2018).

Our results provide the first evidence that maternal exercise can mitigate sevoflurane-induced cognitive impairment in offspring rats. Substantial research, in both humans and animals, has established that maternal exercise during the gestational stage improves cognition in the offspring (Robinson and Bucci, 2012). The offspring of exercised (e.g., treadmill training (Kim et al., 2007; Aksu et al., 2012; Park et al., 2013), swimming (Lee et al., 2006; Marcelino et al., 2013) or voluntary wheel rotation (Robinson and Bucci, 2014)) rodent mothers, particularly, exhibit improved learning and memory when compared with the offspring of sedentary mothers. Several mechanisms have been suggested to account for the beneficial effects of maternal exercise, including increased neurogenesis, long-term potentiation, levels of neurotrophic factors and reduction of oxidative stress in the offspring. Epigenetics has attracted increasing attention in the context of physical exercise-induced alterations in the central nervous system. For example, treadmill exercise alters HATs and HDACs activities, leading to selective changes in hippocampal histone acetylation in rats (Elsner et al., 2011; Spindler et al., 2014). The epigenetic mechanisms involved in the effects of maternal exercise, however, remain unclear. Our data demonstrate that maternal exercise rescued the cognitive deficits caused by repeated prenatal exposure to sevoflurane. This finding is consistent with previous studies, which showed that maternal physical activity attenuated postnatal memory deficits induced by maternal deprivation (Neves et al., 2015), mild chronic postnatal hypoxia (Akhavan et al., 2012) or hyperthermia-induced neuronal apoptosis (Lee et al., 2011) and in rat pups born to lipopolysaccharide-exposed dams or to maternal rats with hypothyroidism (Kim et al., 2015; Shafiee et al., 2016). In the present study, we found that maternal treadmill exercise was able to induce hippocampal histone acetylation at H3K14 and H3K27 in the pups. The up-regulation of BDNF that accompanied up-regulation of histone acetylation indicates that the promoters of BDNF might be acetylated in our maternal exercise model.

**Figure 11:**
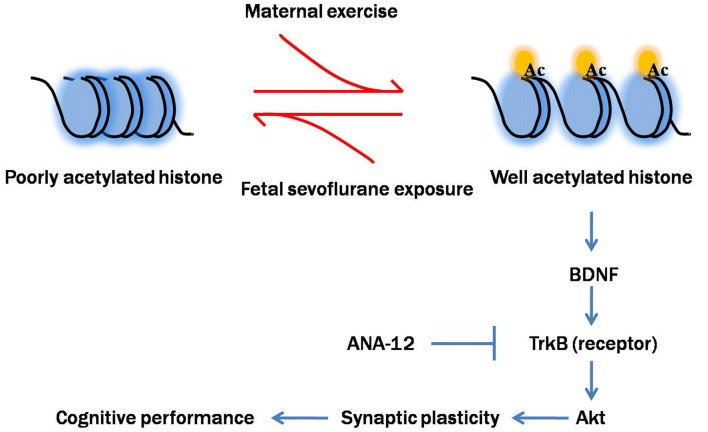
Proposed mechanism of maternal exercise attenuated neurocognitive impairment in offspring born to mothers exposed to sevoflurane during mid-pregnancy. Fetal sevoflurane exposure causes histone hypoacetylation, an epigenetic change that leads to more condensed chromatin structure less conducive to transcription of the target genes BDNF. BDNF is crucial for synaptic plasticity. BDNF binds to TrkB, activating downstream Akt signal pathway. Maternal exercise triggers histone acetylation and activates BDNF/TrkB/Akt signaling. Maternal exercise has neuroprotective effects in mitigating sevoflurane-induced impairments on dendritic spine formation and cognitive functions. Maternal infusion of ANA-12 reverses the beneficial effects of exercise.

To better understand how reduced BDNF expression contributes to sevoflurane-induced cognitive impairments in offspring, we examined the changes in one key component of BDNF-regulated signaling cascades, Akt. Akt is a serine/threonine kinase and plays a critical role in the modulation of cell survival, neurogenesis and synaptic plasticity (Burke, 2007). Previous studies suggested that physical exercise protected against brain injury via activation of Akt signal pathway (Ji et al., 2014; Jung and Kim, 2017). Our results confirmed that disruption of the BDNF/TrkB pathway and downstream Akt signaling cascades not only contributed to sevoflurane-induced cognitive impairments but also played an important role in mediating the beneficial effects of maternal exercise on cognitive function in offspring rats. Notably, we blocked the action of BDNF on TrkB using ANA-12, a selective antagonist for the TrkB receptor family, during maternal treadmill exercise. We found that injection of ANA-12 abolished the increase in p-TrkB and p-Akt levels induced by maternal exercise. Blockade of TrkB receptors also abolished the beneficial effects of maternal exercise on the pups’ learning ability, suggesting that the BDNF/TrkB-stimulated intracellular signaling pathway is critically involved in maternal exercise-induced improved cognition performance in offspring. In addition to TrkB, p75 neurotrophin receptor (p75NTR) is another transmembrane receptor for BDNF, albeit with a low-affinity. However, p75NTR is the high affinity receptor for the precursor of BDNF (proBDNF, immature form of BDNF). The interaction with p75 is still to be determined and it is common considered that p75NTR signaling mainly mediate negative effects such as neuronal apoptosis and long-term depression (Leal et al., 2017). Based on our findings, we propose a cascade of events initiated by sevoflurane-induced neurotoxicity and maternal exercise-induced neuroprotection (Figure 11).

Importantly, sevoflurane treatment did not affect pregnancy outcomes, including: (i) duration of the pregnancy; (ii) size and compositions of the litters; and (iii) body weight gain of pups during lactation. Additionally, body weight gain in the pregnant dams was not affected by maternal exercise protocols.

Our studies have several important limitations. First, to minimize the number of rats used, we did not include an unanesthetized maternal exercise group. However, prior to this study, offspring of pregnant rats that underwent almost the same exercise protocol showed beneficial effects (Gomes da Silva et al., 2016). Second, to avoid the influence of the estrous cycle on the rats’ behavior, we carried out behavioral tests only on male pups. Since previous studies have shown that there may be sex differences in the vulnerability to neurotoxicity caused by anesthetics (Rothstein et al., 2008; Chung et al., 2017), it may be difficult to draw any generalized conclusions. Third, because acetylation of other lysine residues was not assessed and genome-wide analysis of epigenetic modifications was not carried out after sevoflurane exposure, it is possible that we missed dysregulation of histone acetylation at other specific lysine sites. Fourth, we did not evaluate the interaction between H3K14 acetylation or H3K27 acetylation and transcriptional activity of target genes by techniques such as chromatin immunoprecipitation. Additional experimental and mechanistic studies are required to identify the specific effects of H3K14/27 acetylation on regulation of expression of memory-related genes during learning and memory formation, as well as their involvement in memory impairment induced by *in utero* sevoflurane exposure and the neuroprotective effects of maternal exercise. Finally, BDNF binds to TrkB and leads to activation of various signaling modules. We only access the alterations in the levels of p-Akt/Akt, but not other downstream signaling modules like extracellular signal-regulated kinase (ERK; Ji et al., 2014).

## Conclusion

The immediate implication of our results is that a single exposure to 3% sevoflurane produces no long-term cognitive impairment whereas repeated exposure is sufficient to cause long-term cognitive impairment in offspring rats. Repeated exposure also reduces histone acetylation, BDNF signaling and development of dendritic spines in the hippocampus. Maternal exercise attenuated these detrimental effects through enhanced histone acetylation and activation of downstream BDNF/TrkB/Akt signaling.

## Author Contributions

ZW and PZ conceived and designed experiments. ZW, XL and YZ performed experiments, generated and analyzed the data. ZW wrote the manuscript with the help of DT and LW. All authors read and approved the final manuscript.

## Conflict of Interest Statement

The authors declare that the research was conducted in the absence of any commercial or financial relationships that could be construed as a potential conflict of interest.

## Supplementary Material

FIGURE S1 Suspension test of the behavior tests. Tests were performed during P28–32 (*n* = 12/group). Values are mean ± S.E.M. There were no statistical differences between control and maternal sevoflurane exposure groups. Independent *t*-test was used for data analysis.

FIGURE S2 Effects of fetal exposure to sevoflurane on expression of PSD95 and GAP43 in the fetal brain tissues after anesthesia. Fetal brain tissues were harvested at 6 h after anesthesia (*n* = 6/group). **(A)** Representative western blotting images. **(B)** Quantitative analysis of GAP43. **(C)** Quantitative analysis of PSD95. Gapdh was run as an internal standard. Values are mean ± S.E.M. **p* < 0.05, compared with Ctrl×3 group. Independent *t*-test or Mann-Whitney U test was used for data analysis.

FIGURE S3 Body weight gains of dams between G1 until G21. Values are mean ± S.E.M. (*n* = 3). There were no statistical differences among groups. Two-way analysis of variance for repeated measurements followed by Tukey post test was used for data analysis.

TABLE S1 Test used for data analysis.

## References

[B1] Akhavan M. M. Foroutan T. Safari M. Sadighi-Moghaddam B. Emami-Abarghoie M. Rashidy-Pour A. (2012). Prenatal exposure to maternal voluntary exercise during pregnancy provides protection against mild chronic postnatal hypoxia in rat offspring. Pak. J. Pharm. Sci. 25, 233–238. 22186335

[B2] Aksu I. Baykara B. Ozbal S. Cetin F. Sisman A. R. Dayi A.. (2012). Maternal treadmill exercise during pregnancy decreases anxiety and increases prefrontal cortex VEGF and BDNF levels of rat pups in early and late periods of life. Neurosci. Lett. 516, 221–225. 10.1016/j.neulet.2012.03.091 22503727

[B3] Amrock L. G. Starner M. L. Murphy K. L. Baxter M. G. (2015). Long-term effects of single or multiple neonatal sevoflurane exposures on rat hippocampal ultrastructure. Anesthesiology 122, 87–95. 10.1097/ALN.0000000000000477 25289484

[B4] Andropoulos D. B. Greene M. F. (2017). Anesthesia and developing brains—implications of the FDA warning. N. Engl. J. Med. 376, 905–907. 10.1056/NEJMp1700196 28177852

[B5] Barton K. Nickerson J. P. Higgins T. Williams R. K. (2018). Pediatric anesthesia and neurotoxicity: what the radiologist needs to know. Pediatr. Radiol. 48, 31–36. 10.1007/s00247-017-3871-4 28470388

[B6] Bonnaud E. M. Suberbielle E. Malnou C. E. (2016). Histone acetylation in neuronal (dys)function. Biomol. Concepts 7, 103–116. 10.1515/bmc-2016-0002 27101554

[B7] Bredy T. W. Wu H. Crego C. Zellhoefer J. Sun Y. E. Barad M. (2007). Histone modifications around individual BDNF gene promoters in prefrontal cortex are associated with extinction of conditioned fear. Learn. Mem. 14, 268–276. 10.1101/lm.500907 17522015 PMC2216532

[B8] Burke R. E. (2007). Inhibition of mitogen-activated protein kinase and stimulation of Akt kinase signaling pathways: two approaches with therapeutic potential in the treatment of neurodegenerative disease. Pharmacol. Ther. 114, 261–277. 10.1016/j.pharmthera.2007.02.002 17399794 PMC1964795

[B9] Chen K. W. Chen L. (2017). Epigenetic regulation of BDNF gene during development and diseases. Int. J. Mol. Sci. 18:E571. 10.3390/ijms18030571 28272318 PMC5372587

[B10] Chung W. Ryu M. J. Heo J. Y. Lee S. Yoon S. Park H.. (2017). Sevoflurane exposure during the critical period affects synaptic transmission and mitochondrial respiration but not long-term behavior in mice. Anesthesiology 126, 288–299. 10.1097/ALN.0000000000001470 27922840

[B11] Elsner V. R. Lovatel G. A. Bertoldi K. Vanzella C. Santos F. M. Spindler C.. (2011). Effect of different exercise protocols on histone acetyltransferases and histone deacetylases activities in rat hippocampus. Neuroscience 192, 580–587. 10.1016/j.neuroscience.2011.06.066 21745541

[B12] Fan D. Li J. Zheng B. Hua L. Zuo Z. (2016). Enriched environment attenuates surgery-induced impairment of learning, memory and neurogenesis possibly by preserving BDNF expression. Mol. Neurobiol. 53, 344–354. 10.1007/s12035-014-9013-1 25432890

[B13] Fang F. Song R. Ling X. Peng M. Xue Z. Cang J. (2017). Multiple sevoflurane anesthesia in pregnant mice inhibits neurogenesis of fetal hippocampus via repressing transcription factor Pax6. Life Sci. 175, 16–22. 10.1016/j.lfs.2017.03.003 28279665

[B14] Fodale V. Tripodi V. F. Penna O. Fama F. Squadrito F. Mondello E.. (2017). An update on anesthetics and impact on the brain. Expert Opin. Drug Saf. 16, 997–1008. 10.1080/14740338.2017.1351539 28697315

[B15] Gomes da Silva S. de Almeida A. A. Fernandes J. Lopim G. M. Cabral F. R. Scerni D. A.. (2016). Maternal exercise during pregnancy increases BDNF levels and cell numbers in the hippocampal formation but not in the cerebral cortex of adult rat offspring. PLoS One 11:e0147200. 10.1371/journal.pone.0147200 26771675 PMC4714851

[B16] Jakovcevski M. Akbarian S. (2012). Epigenetic mechanisms in neurological disease. Nat. Med. 18, 1194–1204. 10.1038/nm.2828 22869198 PMC3596876

[B17] Ji J. F. Ji S. J. Sun R. Li K. Zhang Y. Zhang L. Y.. (2014). Forced running exercise attenuates hippocampal neurogenesis impairment and the neurocognitive deficits induced by whole-brain irradiation via the BDNF-mediated pathway. Biochem. Biophys. Res. Commun. 443, 646–651. 10.1016/j.bbrc.2013.12.031 24333433

[B18] Jia M. Liu W. X. Sun H. L. Chang Y. Q. Yang J. J. Ji M. H.. (2015). Suberoylanilide hydroxamic acid, a histone deacetylase inhibitor, attenuates postoperative cognitive dysfunction in aging mice. Front. Mol. Neurosci. 8:52. 10.3389/fnmol.2015.00052 26441515 PMC4585136

[B19] Jia M. Liu W. X. Yang J. J. Xu N. Xie Z. M. Ju L. S.. (2016). Role of histone acetylation in long-term neurobehavioral effects of neonatal Exposure to sevoflurane in rats. Neurobiol. Dis. 91, 209–220. 10.1016/j.nbd.2016.03.017 27001149 PMC4860151

[B20] Jung S. Y. Kim D. Y. (2017). Treadmill exercise improves motor and memory functions in cerebral palsy rats through activation of PI3K-Akt pathway. J. Exerc. Rehabil. 13, 136–142. 10.12965/jer.1734964.482 28503524 PMC5412485

[B21] Kim H. Lee S. H. Kim S. S. Yoo J. H. Kim C. J. (2007). The influence of maternal treadmill running during pregnancy on short-term memory and hippocampal cell survival in rat pups. Int. J. Dev. Neurosci. 25, 243–249. 10.1016/j.ijdevneu.2007.03.003 17434282

[B22] Kim K. Sung Y. H. Seo J. H. Lee S. W. Lim B. V. Lee C. Y.. (2015). Effects of treadmill exercise-intensity on short-term memory in the rats born of the lipopolysaccharide-exposed maternal rats. J. Exerc. Rehabil. 11, 296–302. 10.12965/jer.150264 26730379 PMC4697777

[B23] Kong F. J. Ma L. L. Hu W. W. Wang W. N. Lu H. S. Chen S. P. (2012). Fetal exposure to high isoflurane concentration induces postnatal memory and learning deficits in rats. Biochem. Pharmacol. 84, 558–563. 10.1016/j.bcp.2012.06.001 22705347

[B24] Koppel I. Timmusk T. (2013). Differential regulation of Bdnf expression in cortical neurons by class-selective histone deacetylase inhibitors. Neuropharmacology 75, 106–115. 10.1016/j.neuropharm.2013.07.015 23916482

[B25] Kowianski P. Lietzau G. Czuba E. Waskow M. Steliga A. Morys J. (2018). BDNF: a key factor with multipotent impact on brain signaling and synaptic plasticity. Cell. Mol. Neurobiol. 38, 579–593. 10.1007/s10571-017-0510-4 28623429 PMC5835061

[B26] Leal G. Bramham C. R. Duarte C. B. (2017). BDNF and hippocampal synaptic plasticity. Vitam. Horm. 104, 153–195. 10.1016/bs.vh.2016.10.004 28215294

[B28] Lee J. Cho J. Y. Oh S. D. Kim S. M. Shim Y. T. Park S.. (2011). Maternal exercise reduces hyperthermia-induced apoptosis in developing mouse brain. Int. J. Hyperthermia 27, 445–452. 10.3109/02656736.2011.569967 21756042

[B29] Lee S. Chung W. Park H. Park H. Yoon S. Park S.. (2017). Single and multiple sevoflurane exposures during pregnancy and offspring behavior in mice. Paediatr. Anaesth. 27, 742–751. 10.1111/pan.13139 28497474

[B27] Lee H. H. Kim H. Lee J. W. Kim Y. S. Yang H. Y. Chang H. K.. (2006). Maternal swimming during pregnancy enhances short-term memory and neurogenesis in the hippocampus of rat pups. Brain Dev. 28, 147–154. 10.1016/j.braindev.2005.05.007 16368211

[B30] Liang B. Fang J. (2016). Postnatal isoflurane exposure induces cognitive impairment and abnormal histone acetylation of glutamatergic systems in the hippocampus of adolescent rats. J. Mol. Neurosci. 60, 11–20. 10.1007/s12031-016-0756-1 27307148

[B31] Lu H. Liufu N. Dong Y. Xu G. Zhang Y. Shu L.. (2017). Sevoflurane acts on ubiquitination-proteasome pathway to reduce postsynaptic density 95 protein levels in young mice. Anesthesiology 127, 961–975. 10.1097/ALN.0000000000001889 28968276 PMC5685882

[B32] Majumdar D. Nebhan C. A. Hu L. Anderson B. Webb D. J. (2011). An APPL1/Akt signaling complex regulates dendritic spine and synapse formation in hippocampal neurons. Mol. Cell. Neurosci. 46, 633–644. 10.1016/j.mcn.2011.01.003 21236345 PMC3046229

[B33] Marcelino T. B. Longoni A. Kudo K. Y. Stone V. Rech A. de Assis A. M.. (2013). Evidences that maternal swimming exercise improves antioxidant defenses and induces mitochondrial biogenesis in the brain of young Wistar rats. Neuroscience 246, 28–39. 10.1016/j.neuroscience.2013.04.043 23639877

[B34] Martínez-Levy G. A. Cruz-Fuentes C. S. (2014). Genetic and epigenetic regulation of the brain-derived neurotrophic factor in the central nervous system. Yale J. Biol. Med. 87, 173–186. 24910563 PMC4031791

[B35] Murphy K. L. Baxter M. G. (2013). Long-term effects of neonatal single or multiple isoflurane exposures on spatial memory in rats. Front. Neurol. 4:87. 10.3389/fneur.2013.00087 23847588 PMC3703565

[B36] Nakai T. Nagai T. Tanaka M. Itoh N. Asai N. Enomoto A.. (2014). Girdin phosphorylation is crucial for synaptic plasticity and memory: a potential role in the interaction of BDNF/TrkB/Akt signaling with NMDA receptor. J. Neurosci. 34, 14995–15008. 10.1523/JNEUROSCI.2228-14.2014 25378165 PMC6608366

[B37] Neves B. H. Menezes J. Souza M. A. Mello-Carpes P. B. (2015). Physical exercise prevents short and long-term deficits on aversive and recognition memory and attenuates brain oxidative damage induced by maternal deprivation. Physiol. Behav. 152, 99–105. 10.1016/j.physbeh.2015.09.019 26403760

[B38] Olutoye O. A. Sheikh F. Zamora I. J. Yu L. Akinkuotu A. C. Adesina A. M.. (2016). Repeated isoflurane exposure and neuroapoptosis in the midgestation fetal sheep brain. Am. J. Obstet. Gynecol. 214, 542.e1–542.e8. 10.1016/j.ajog.2015.10.927 26546852

[B39] Palanisamy A. (2012). Maternal anesthesia and fetal neurodevelopment. Int. J. Obstet. Anesth. 21, 152–162. 10.1016/j.ijoa.2012.01.005 22405978

[B40] Park J. W. Kim M. H. Eo S. J. Lee E. H. Kang J. S. Chang H. K.. (2013). Maternal exercise during pregnancy affects mitochondrial enzymatic activity and biogenesis in offspring brain. Int. J. Neurosci. 123, 253–264. 10.3109/00207454.2012.755969 23227820

[B41] Peixoto L. Abel T. (2013). The role of histone acetylation in memory formation and cognitive impairments. Neuropsychopharmacology 38, 62–76. 10.1038/npp.2012.86 22669172 PMC3521994

[B42] Qureshi I. A. Mehler M. F. (2011). Advances in epigenetics and epigenomics for neurodegenerative diseases. Curr. Neurol. Neurosci. Rep. 11, 464–473. 10.1007/s11910-011-0210-2 21671162 PMC4461866

[B43] Robinson A. M. Bucci D. J. (2012). Maternal exercise and cognitive functions of the offspring. Cogn. Sci. 7, 187–205. 26664667 PMC4671504

[B44] Robinson A. M. Bucci D. J. (2014). Physical exercise during pregnancy improves object recognition memory in adult offspring. Neuroscience 256, 53–60. 10.1016/j.neuroscience.2013.10.012 24157927 PMC3874249

[B45] Rothstein S. Simkins T. Nuñez J. L. (2008). Response to neonatal anesthesia: effect of sex on anatomical and behavioral outcome. Neuroscience 152, 959–969. 10.1016/j.neuroscience.2008.01.027 18329814 PMC2396530

[B46] Shafiee S. M. Vafaei A. A. Rashidy-Pour A. (2016). Effects of maternal hypothyroidism during pregnancy on learning, memory and hippocampal BDNF in rat pups: beneficial effects of exercise. Neuroscience 329, 151–161. 10.1016/j.neuroscience.2016.04.048 27181637

[B47] Silbereis J. C. Pochareddy S. Zhu Y. Li M. Sestan N. (2016). The cellular and molecular landscapes of the developing human central nervous system. Neuron 89, 248–268. 10.1016/j.neuron.2015.12.008 26796689 PMC4959909

[B48] Spindler C. Cechinel L. R. Basso C. Moyses F. Bertoldi K. Roesler R.. (2014). Treadmill exercise alters histone acetyltransferases and histone deacetylases activities in frontal cortices from wistar rats. Cell. Mol. Neurobiol. 34, 1097–1101. 10.1007/s10571-014-0096-z 25149076 PMC11488882

[B49] Suehara T. Morishita J. Ueki M. Ueno M. Maekawa N. Mizobuchi S. (2016). Effects of sevoflurane exposure during late pregnancy on brain development of offspring mice. Paediatr. Anaesth. 26, 52–59. 10.1111/pan.12785 26645425

[B50] Wang Y. Cheng Y. Liu G. Tian X. Tu X. Wang J. (2012). Chronic exposure of gestation rat to sevoflurane impairs offspring brain development. Neurol. Sci. 33, 535–544. 10.1007/s10072-011-0762-6 21948083

[B51] Wang Y. Yin S. Xue H. Yang Y. Zhang N. Zhao P. (2018). Mid-gestational sevoflurane exposure inhibits fetal neural stem cell proliferation and impairs postnatal learning and memory function in a dose-dependent manner. Dev. Biol. 435, 185–197. 10.1016/j.ydbio.2018.01.022 29410165

[B52] Workman A. D. Charvet C. J. Clancy B. Darlington R. B. Finlay B. L. (2013). Modeling transformations of neurodevelopmental sequences across mammalian species. J. Neurosci. 33, 7368–7383. 10.1523/JNEUROSCI.5746-12.2013 23616543 PMC3928428

[B53] Xu Y. Tian Y. Tian Y. Li X. Zhao P. (2016). Autophagy activation involved in hypoxic-ischemic brain injury induces cognitive and memory impairment in neonatal rats. J. Neurochem. 139, 795–805. 10.1111/jnc.13851 27659442

[B54] Zhang J. Dong Y. Zhou C. Zhang Y. Xie Z. (2015). Anesthetic sevoflurane reduces levels of hippocalcin and postsynaptic density protein 95. Mol. Neurobiol. 51, 853–863. 10.1007/s12035-014-8746-1 24870966

[B55] Zheng H. Dong Y. Xu Z. Crosby G. Culley D. J. Zhang Y.. (2013). Sevoflurane anesthesia in pregnant mice induces neurotoxicity in fetal and offspring mice. Anesthesiology 118, 516–526. 10.1097/ALN.0b013e3182834d5d 23314109 PMC3580035

