# Supplementary figures and images for: Effects of Sevoflurane Exposure During Mid-Pregnancy on Learning and Memory in Offspring Rats: Beneficial Effects of Maternal Exercise
Source: Front Cell Neurosci. 2018 May 3;12:122. doi: 10.3389/fncel.2018.00122 (PMC5943573; doi:10.3389/fncel.2018.00122)

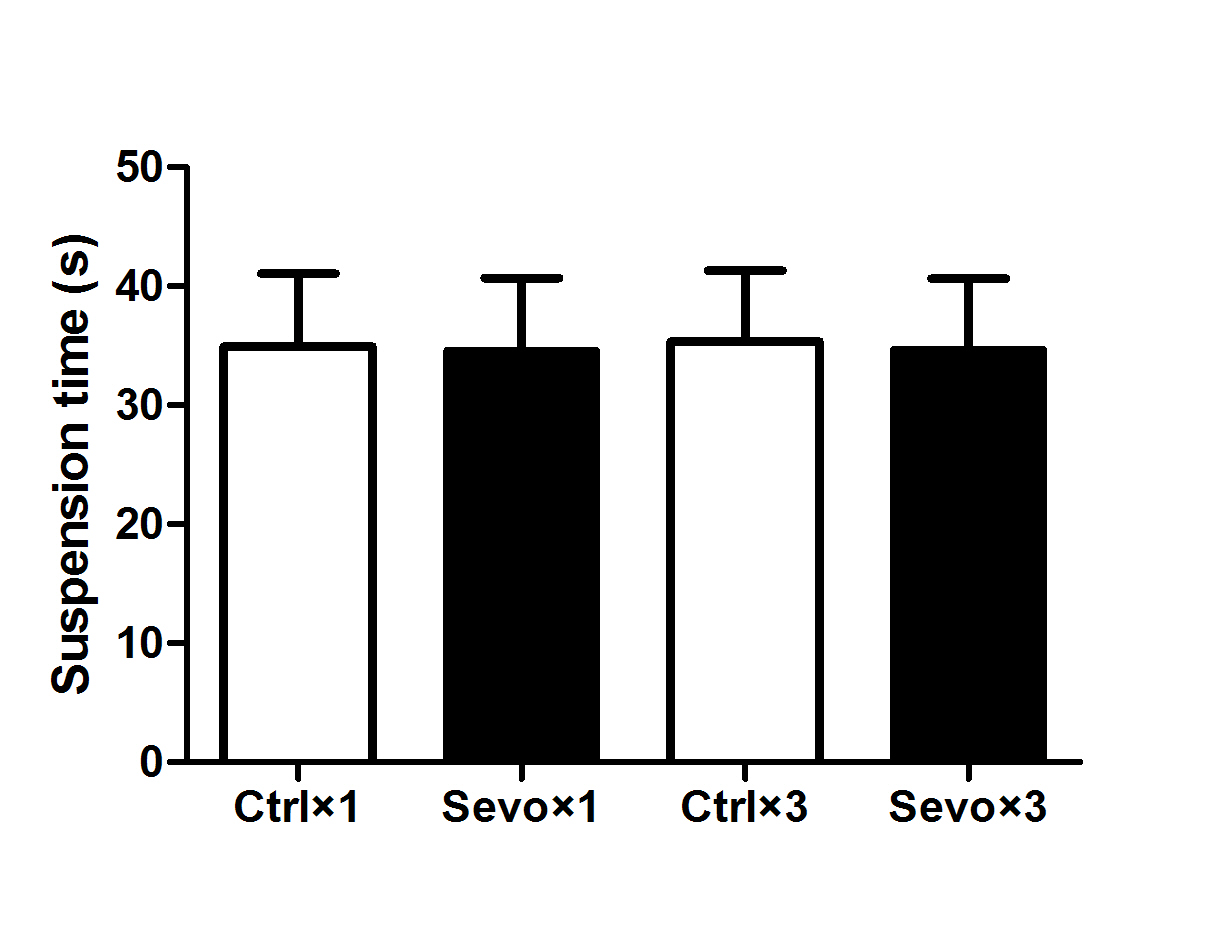

Supplement: FIGURE S1 — Suspension test of the behavior tests. Tests were performed during P28–32 (n = 12/group). Values are mean ± S.E.M. There were no statistical differences between control and maternal sevoflurane exposure groups. Independent t-test was used for data analysis. [file Image_1.JPEG]

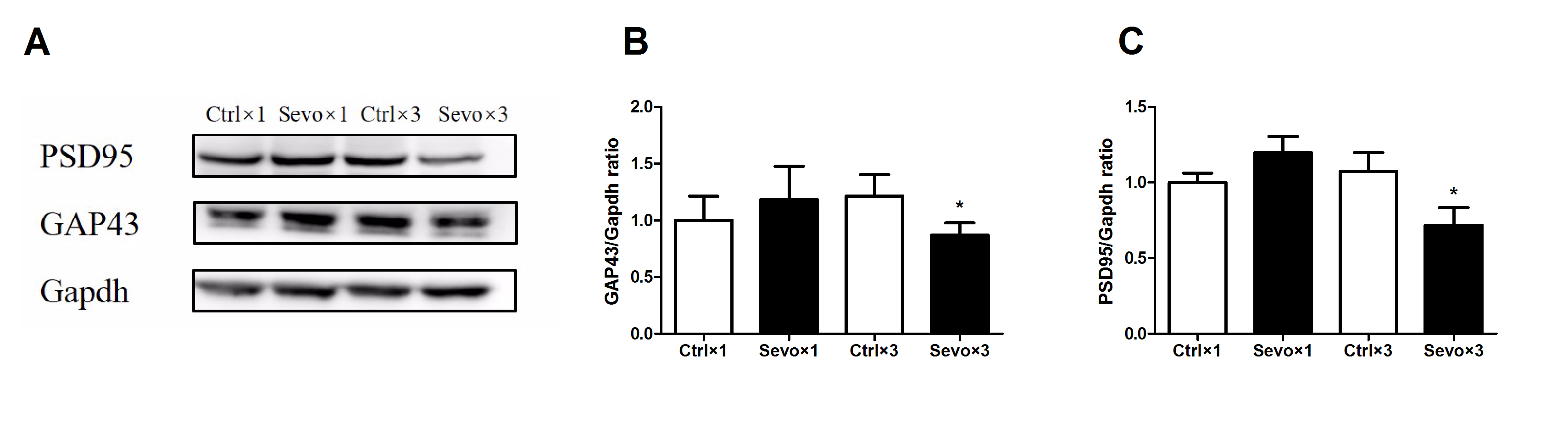

Supplement: FIGURE S2 — Effects of fetal exposure to sevoflurane on expression of PSD95 and GAP43 in the fetal brain tissues after anesthesia. Fetal brain tissues were harvested at 6 h after anesthesia (n = 6/group). (A) Representative western blotting images. (B) Quantitative analysis of GAP43. (C) Quantitative analysis of PSD95. Gapdh was run as an internal standard. Values are mean ± S.E.M. *p < 0.05, compared with Ctrl×3 group. Independent t-test or Mann-Whitney U test was used for data analysis. [file Image_2.jpg]

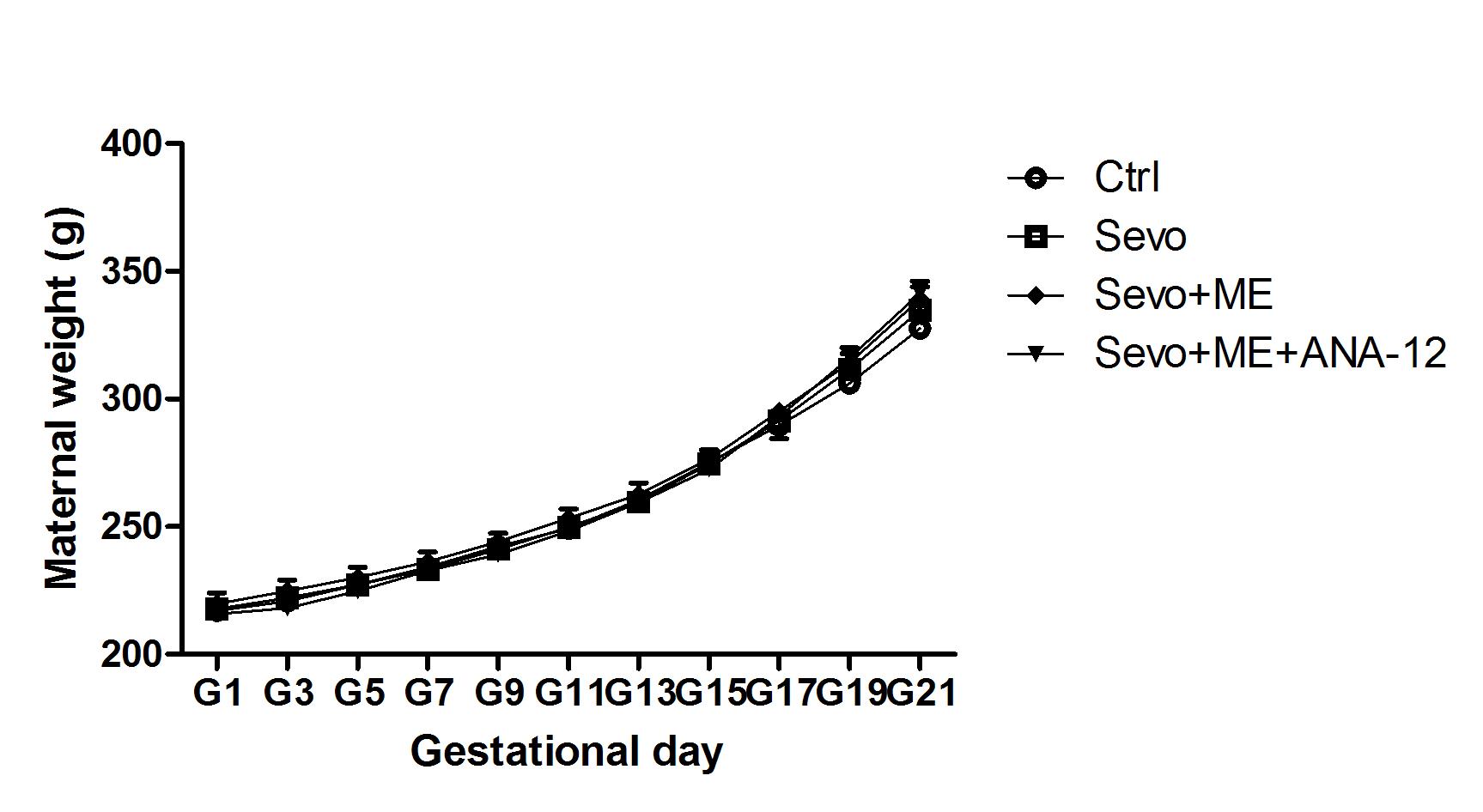

Supplement: FIGURE S3 — Body weight gains of dams between G1 until G21. Values are mean ± S.E.M. (n = 3). There were no statistical differences among groups. Two-way analysis of variance for repeated measurements followed by Tukey post test was used for data analysis. [file Image_3.JPEG]
