# Supplementary material for: Effects of Sevoflurane Exposure During Mid-Pregnancy on Learning and Memory in Offspring Rats: Beneficial Effects of Maternal Exercise
Source: Front Cell Neurosci. 2018 May 3;12:122. doi: 10.3389/fncel.2018.00122 (PMC5943573; doi:10.3389/fncel.2018.00122)
Supplement: TABLE S1 — Test used for data analysis. [file Table_1.DOC]

Supplementary Table 1 | Test used for data analysis

| Figure | Parameter | Statistical test |
| --- | --- | --- |
| 2B | Ace-H3K9/H3 ratio | 1. Ctrl×1 vs Sevo×1 group:  6h, 24h, P0: Independent t-test  2. Ctrl×3 vs Sevo×3 group:  6h, 24h: Independent t-test  P0: Mann-Whitney U test |
| 2C | Ace-H3K14/H3 ratio | 1. Ctrl×1 vs Sevo×1 group:  6h, 24h, P0: Independent t-test  2. Ctrl×3 vs Sevo×3 group:  6h: Welch’s t-test  24h, P0: Independent t-test |
| 2D | Ace-H3K27/H3 ratio | 1. Ctrl×1 vs Sevo×1 group:  6h: Welch’s t-test  24h, P0: Independent t-test  2. Ctrl×3 vs Sevo×3 group:  6h, 24h: Independent t-test  P0: Welch’s t-test |
| 2E | BDNF/Gapdh ratio | 1. Ctrl×1 vs Sevo×1 group:  6h, 24h, P0: Independent t-test  2. Ctrl×3 vs Sevo×3 group:  6h, P0: Independent t-test  24h: Welch’s t-test |
| 3A | The average swimming speed (cm/s) | 1. Ctrl×1 vs Sevo×1 group:  Independent t-test  2. Ctrl×3 vs Sevo×3 group:  Independent t-test |
| 3B | Escape latency (s) | 1. Ctrl×1 vs Sevo×1 group:  Two-way analysis of variance for repeated measurements, followed by Tukey post test  2. Ctrl×3 vs Sevo×3 group:  Two-way analysis of variance for repeated measurements, followed by Tukey post test |
| 3C | Platform crossing time | 1. Ctrl×1 vs Sevo×1 group:  Mann-Whitney U test  2. Ctrl×3 vs Sevo×3 group:  Mann-Whitney U test |
| 3D | Time spent in quadrant 1 (s) | 1. Ctrl×1 vs Sevo×1 group:  Mann-Whitney U test  2. Ctrl×3 vs Sevo×3 group:  Independent t-test |
|  | Time spent in quadrant 2 (s) | 1. Ctrl×1 vs Sevo×1 group:  Independent t-test  2. Ctrl×3 vs Sevo×3 group:  Mann-Whitney U test |
|  | Time spent in quadrant 3(s) | 1. Ctrl×1 vs Sevo×1 group:  Independent t-test  2. Ctrl×3 vs Sevo×3 group:  Independent t-test |
|  | Time spent in quadrant 4(s) | 1. Ctrl×1 vs Sevo×1 group:  Mann-Whitney U test  2. Ctrl×3 vs Sevo×3 group:  Mann-Whitney U test |
| 4B | PSD/Gapdh ratio | 1. Ctrl×1 vs Sevo×1 group:  Independent t-test  2. Ctrl×3 vs Sevo×3 group:  Independent t-test |
| 4C | GAP-43/Gapdh ratio | 1. Ctrl×1 vs Sevo×1 group:  Independent t-test  2. Ctrl×3 vs Sevo×3 group:  Independent t-test |
| 5B | Neuronal density ratio | 1. Ctrl×1 vs Sevo×1 group:  Independent t-test  2. Ctrl×3 vs Sevo×3 group:  Independent t-test |
| 5D | The number of dendritic spines/10 μm | 1. Ctrl×1 vs Sevo×1 group:  Independent t-test  2. Ctrl×3 vs Sevo×3 group:  Independent t-test |
| 6B | Ace-H3K14/H3 ratio | One-way analysis of variance, followed by Tukey post hoc multiple comparison tests |
| 6D | Ace-H3K27/H3 ratio | One-way analysis of variance, followed by Tukey post hoc multiple comparison tests |
| 6F | BDNF/Gapdh ratio | One-way analysis of variance, followed by Tukey post hoc multiple comparison tests |
| 7A | The average swimming speed (cm/s) | Kruskal-Wallis H test |
| 7B | Escape latency (s) | Two-way analysis of variance for repeated measurements, followed by Tukey post test |
| 7C | Platform crossing time | Two-way analysis of variance for repeated measurements, followed by Tukey post test |
| 7D | Time spent in quadrant 1 (s) | Two-way analysis of variance for repeated measurements, followed by Tukey post test |
|  | Time spent in quadrant 2 (s) | Two-way analysis of variance for repeated measurements, followed by Tukey post test |
|  | Time spent in quadrant 3 (s) | Two-way analysis of variance for repeated measurements, followed by Tukey post test |
|  | Time spent in quadrant 4 (s) | Kruskal-Wallis H test |
| 8B | Neuronal density ratio | One-way analysis of variance, followed by Tukey post hoc multiple comparison tests |
| 8D | The number of dendritic spines/10 μm | One-way analysis of variance, followed by Tukey post hoc multiple comparison tests |
| 9B | PSD/Gapdh ratio | One-way analysis of variance, followed by Tukey post hoc multiple comparison tests |
| 9C | GAP-43/Gapdh ratio | One-way analysis of variance, followed by Tukey post hoc multiple comparison tests |
| 10B | P-TrkB/TrkB ratio | One-way analysis of variance, followed by Tukey post hoc multiple comparison tests |
| 10D | P-Akt/Akt ratio | One-way analysis of variance, followed by Tukey post hoc multiple comparison tests |
| SUPPLEMENTARY FIGURE 1 | Time spent in suspension test (s) | 1. Ctrl×1 vs Sevo×1 group:  Independent t-test  2. Ctrl×3 vs Sevo×3 group:  Independent t-test |
| SUPPLEMENTARY FIGURE 2 B | 1.PSD/Gapdh ratio | 1. Ctrl×1 vs Sevo×1 group:  Independent t-test  2. Ctrl×3 vs Sevo×3 group:  Mann-Whitney U test |
|  | 2.GAP-43/Gapdh ratio | 1. Ctrl×1 vs Sevo×1 group:  Independent t-test  2. Ctrl×3 vs Sevo×3 group:  Independent t-test |
| SUPPLEMENTARY FIGURE 3 | Weight (g) | Two-way analysis of variance for repeated measurements, followed by Tukey post test |
